# Study protocol: implementing and evaluating a trauma-informed model of care in residential youth treatment for substance use disorders

**DOI:** 10.3389/fpsyt.2023.1169794

**Published:** 2023-09-27

**Authors:** Zoe C. Walter, Molly Carlyle, Nick Kerswell, Valeriya Mefodeva, Reg D. V. Nixon, Vanessa E. Cobham, Leanne Hides

**Affiliations:** ^1^School of Psychology, Faculty of Health and Behavioral Sciences, University of Queensland, Brisbane, QLD, Australia; ^2^National Center for Youth Substance Use Research, Faculty of Health and Behavioral Sciences, University of Queensland, Brisbane, QLD, Australia; ^3^Lives Lived Well, Brisbane, QLD, Australia; ^4^Flinders University Institute for Mental Health and Wellbeing, Flinders University, Adelaide, SA, Australia; ^5^College of Education, Psychology and Social Work, Flinders University, Adelaide, SA, Australia; ^6^Children’s Health Queensland, Child and Youth Mental Health Service, Brisbane, QLD, Australia

**Keywords:** trauma-informed care, alcohol and other drug use, trauma, implementation, residential rehabilitation

## Abstract

**Introduction:**

Comorbidity between Substance Use Disorders and trauma/post-traumatic stress disorder (PTSD) is common, particularly within residential treatment services. Comorbidity is associated with poorer treatment retention and treatment outcomes. Integrated treatment approaches are increasingly recommended but are still under examined in residential treatment services. This study will implement and evaluate a novel model of trauma-informed care (TIC) in a youth (18–35 years) residential substance use treatment service.

**Methods and analysis:**

A single-armed, phase 1 implementation trial will be conducted in one residential treatment service. The model, co-developed with staff, incorporates: (i) workforce development in TIC through staff training and clinical supervision; adaptions to the service (ii) policies, procedures, and physical settings and (iii) treatment program adaptions (in delivery style and content) to be more trauma-informed; (iv) client screening and feedback for trauma and PTSD at service entry; and (v) the provision of support, referral and/or trauma-focused therapy to those with PTSD. Service outcomes will include adherence to the TIC model and client treatment completion. Client substance use and mental health measures will be collected at service entry, and 1-, 3-, 6- and 12-months follow up. Staff outcomes, including workplace satisfaction, burnout, and fatigue, as well as perceptions and confidence in delivering TIC will be collected at baseline, and at 3-, 6-, 12- and 18-months following training in the model. The sustainability of the delivery of the TIC model of care will be evaluated for 12 months using service and staff outcomes.

**Ethics and dissemination:**

The study has received ethical approval by the University of Queensland (Approval number: 2020000949). The results will be disseminated through publication in a peer-reviewed scientific journal, presentations at scientific conferences, and distributed via a report and presentations to the partner organization.

**Clinical trial registration**: ACTRN12621000492853.

## Introduction

People with substance use disorders (SUDs) report disproportionately higher rates of lifetime trauma, which can both precede (1–5) and occur as a consequence of substance use (6). Research indicates up to 90% of those seeking help for SUDs report prior trauma (7, 8), which can precipitate post-traumatic stress disorder (PTSD); a psychiatric condition characterized by symptoms of intrusion, avoidance, negative mood and cognitions, hyperarousal, dissociation, and poor functioning (9). In particular, both childhood trauma and associated PTSD increase risk for early substance use, which is associated with greater severity and complexity of SUDs (10, 11). Comorbid SUD and PTSD is reported in 43–50% of individuals seeking SUD treatment (8, 12, 13), and is associated with poorer outcomes than a SUD or PTSD diagnosis alone. This includes a greater risk of disability, poorer physical and mental health (11, 14), and reduced treatment retention (15, 16). Current treatments for SUDs demonstrate limited long-term efficacy for reducing relapse and craving (17–19), and improving mental health outcome among people with comorbid SUD and PTSD (20). As a result, there are increasing calls for SUD treatment settings to offer trauma-informed and trauma-focused care (9, 21). The link between early trauma exposure, early substance use and greater severity of SUD, and the subsequent greater complexity of treatment needs underscores the importance of early intervention and trauma-informed care particularly for young people (22). Trauma-informed care (TIC) refers to service delivery that is grounded in an understanding of how trauma affects peoples’ lives, service needs, and service usage. Trauma-focused approaches provide integrated psychosocial treatment to address symptoms of SUD and PTSD concurrently.

Residential treatment is a common treatment approach for people with SUD and other mental health comorbidities. Services typically provide live-in treatment through structured programs and 24-h support in safe accommodation (23), although the nature and specifics of such services vary widely (24). Implementing TIC in residential treatment may reduce the risk of re-traumatization, enhance individual treatment outcomes, and improve staff confidence with managing trauma (25, 26). As residential treatment encompasses multiple systems of care, in which an individual is situated within a program that sits within a broader environment, integrating trauma informed models of care at both a service level and individual level may be particularly important.

### Trauma-informed care at a service level

An organizational approach to TIC assumes a history of trauma exposure in all clients, whereby there is a sensitivity to trauma at service system levels (27). Based on trauma theory and empirical evidence of trauma-informed practices to design service systems, common frameworks of trauma-informed care propose five essential values of TIC for staff and clients: safety (ensuring all staff and clients feel psychologically safe), trustworthiness (transparent operations and adequate follow through on promised services), choice (clients are given options wherever possible), and collaboration (partnering between clients and staff) through empowerment (reducing the power differentials between clients, staff and supervisors) (26, 28, 29). These guiding principles demonstrate distinct yet related benefits (30), and are measurable across an organization (30, 31). TIC has been implemented in psychiatric (32), justice (33, 34), medical (35), and child/youth welfare systems (36), for both outpatient and residential services. Benefits of TIC include greater staff satisfaction, retention, commitment, and performance (37). For clients, TIC is associated with reduced youth misconduct and increased feelings of safety in juvenile justice systems, reduced disciplinary events in schools, in addition to reduced aggressive patient incidents, restraints, and seclusions in inpatient psychiatric units [see (38), for a review]. However, much of this literature has evaluated outcomes using short follow-up periods and rarely has the sustainability of TIC models been evaluated post implementation (38).

In models of TIC developed for alcohol and drug use settings, trauma is addressed via: (i) routine screening for symptoms of PTSD in clients entering treatment, (ii) providing feedback to clients about their scores and offering appropriate support and/or treatment, (iii) ensuring the service environment is sensitive to trauma and its role in substance use, and (iv) training staff in TIC and providing them with appropriate supervision to identify and manage trauma-related reactions in clients (39). The delivery of integrated TIC substance use treatment can increase treatment retention in adults compared with standard treatment (40), and reduce PTSD symptoms in adolescents (41). However, limited research has implemented TIC in residential services for SUDs or examined sustainability of TIC models in substance use treatment settings.

### Trauma-informed treatment at an individual level

In addition to organizational practice approaches, the implementation of TIC may include individualized and group-based trauma-focused treatment (9). In outpatient settings, trauma-focused individual and group therapies are more effective at reducing both symptoms of PTSD and substance use, when compared to non-trauma therapies (see (42), for a review). One study also reported that trauma-focused therapy was not associated with any greater PTSD symptom exacerbation than relapse prevention (43), supporting the safety and tolerability of addressing trauma within treatment for SUDs.

Trauma-focused treatments typically use cognitive-behavioral principles to promote processing of the traumatic event and its meaning (44). One ‘gold-standard’ therapy for PTSD across several settings and populations is Cognitive Processing Therapy (CPT) (45–51). Two meta-analyses have demonstrated that CPT produces the greatest average effect sizes for improving PTSD outcomes when compared to psychotropic medication and other psychological treatments, including exposure-based interventions (52, 53). The treatment effects of CPT for PTSD have also been shown to be enduring 5 years after treatment (54). Despite this, there has been limited research examining integrated CPT for concurrent PTSD and SUD, with one study using a single case design in an outpatient setting (55, 56). Further exploration on how CPT could be integrated into the treatment of SUDs in residential settings may help to reduce comorbid symptoms of PTSD.

### The current trial

Providing TIC in residential treatment services for young people may help ameliorate problematic substance use and reduce risk of relapse (25) as well as improve symptoms of PTSD (20). The current trial aims to: (i) determine the feasibility of implementing a new model of TIC in a youth (18–35 years) residential substance use treatment service, (ii) evaluate the impact on client, staff, and service outcomes, and (iii) examine the sustainability of the delivery of the TIC model once the formal evaluation ends.

The frameworks used to guide the current project were the “Consolidated Framework for Implementation Research” (CFIR; (57)) and the “Exploration, Preparation, Implementation, Sustainment” [EPIS; (58)] framework. CFIR offers a comprehensive approach to understanding the dynamic factors influencing intervention implementation across individual, team, and organizational levels. This allowed us to systematically assess key constructs, such as intervention characteristics, inner and outer settings, individual characteristics, and implementation process, to inform our implementation strategies. The EPIS framework was used to develop a structured phased approach that provided sequential phases of exploration, preparation, implementation, and sustainment. This combined approach enabled us to better understand the organizational context, stakeholder engagement, and potential barriers and facilitators throughout each planned phase, and provided a structure to develop an intervention with longevity post research involvement.

## Methods and analysis

### Trial design and sample selection

This single-armed, uncontrolled Phase 1 implementation trial will develop and implement a new model of TIC into one residential treatment service in Queensland, Australia and evaluate client, staff, and service outcomes. The service currently provides a live-in six-week treatment program for young people aged 18–35 years with substance use problems, using individual and group-based therapy.

The sample for the study will be clients and staff in the participating residential treatment service, who consent to participate in the TIC evaluation. Staff include counselors/case managers; management; medical staff such as nurses and psychiatry registrars; night and support staff. Non-identifiable service data will also be collected. The Standard Protocol Items for Randomized Trials (SPIRIT) checklist was adhered to during the development of this protocol, see Appendix 1.

#### Patient and public involvement

To assess the specific needs of the residential service, qualitative interviews were conducted with 20 residential staff and 18 clients (59). The development of the research question and outcome measures was informed by this qualitative research. Both staff and clients perceived comorbid SUD and PTSD to be a significant challenge in residential treatment and recognized the need for integrating TIC and PTSD treatment in these settings. However, staff had an inconsistent understanding of what it means for a service to become trauma-informed and reported considerable variability in their practical skills for managing trauma (59). The model was co-designed with residential service clients, staff, and management, based on existing models of TIC that were developed for adult outpatient services (60). Codesign occurred between February and July 2020. After the qualitative interviews and initial consultations, the model was refined in collaboration with residential service clients, staff, and management. There was no patient involvement in the recruitment to and conduct of the study. The trial results will be disseminated in the form of a report to the treatment services.

### The trauma informed care model

**Figure 1:**
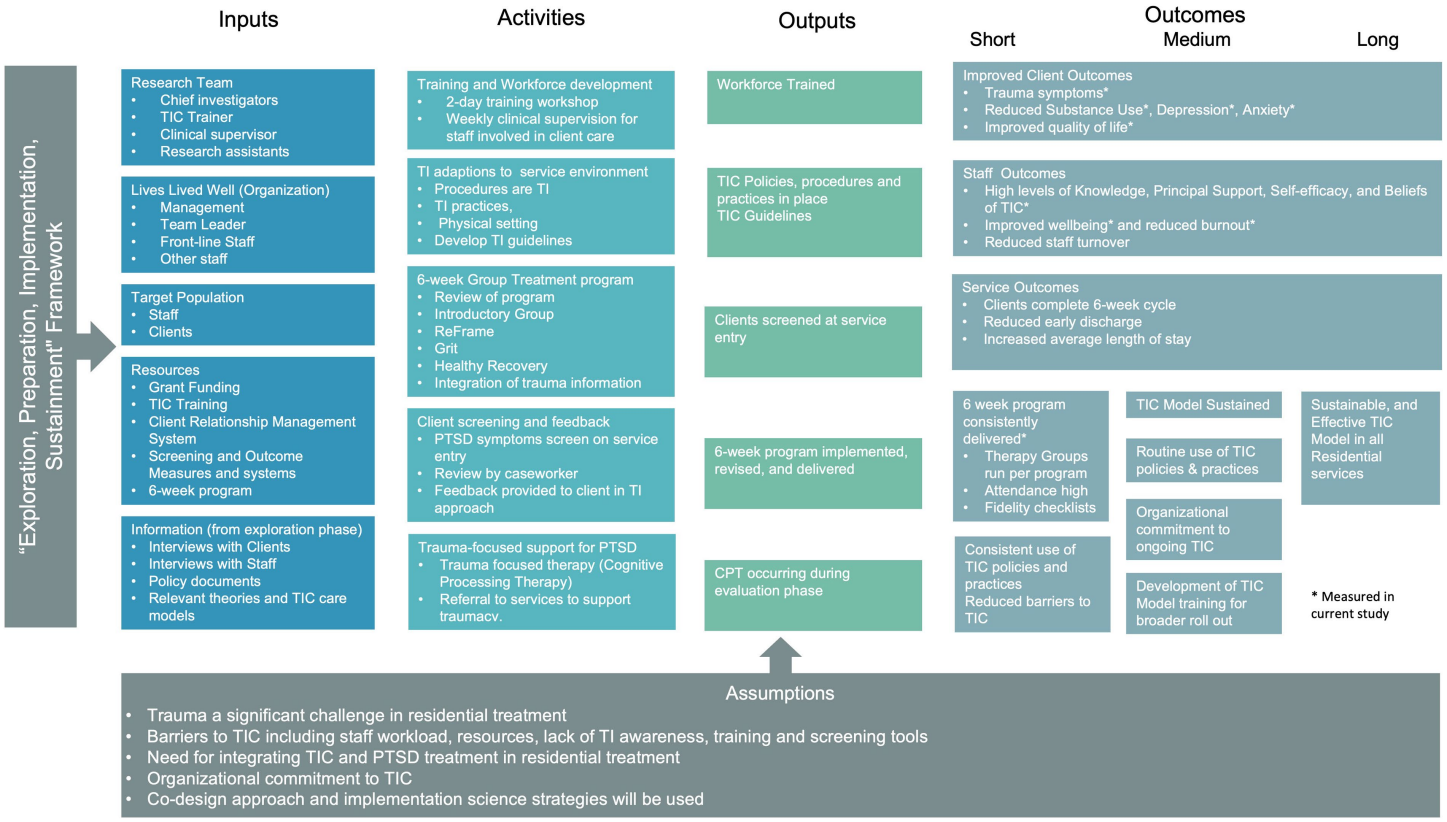
Logic model for the newly developed trauma-informed model of care in residential youth treatment for substance use disorders.

The core components of the new TIC model of care, described below, include: workforce development, adapting the program and broader service environment, screening for trauma, and providing trauma-focused therapy. A Logic Model provides an overview of the development and proposed implementation and outcomes of the TIC model of care (see Figure 1).

#### Workforce development

Following recommendations from research and practice for how to implement TIC models for SUDs (21, 28, 61), workforce development had the following aims: ensure staff working with clients have an awareness of the extent of trauma exposure, increase understanding of the consequences of trauma exposure and its impact on behavior and recovery, facilitate recognition of the signs and symptoms of trauma-related disorders, and develop skills to apply this knowledge in practice using brief intervention strategies (61). A 2-day training workshop for staff was developed and delivered by a clinical psychologist with specialist skills in TIC in August 2020. The aim of the workshop was to build staff knowledge of TIC, and their skills and confidence in trauma screening, feedback, and management of trauma-related behaviors in residential AOD treatment settings. A summary of the workshop content is provided in Table 1.

**Table 1:** Essential areas of trauma-informed care included in the staff training.

Topic	Details
*Introductory skills in trauma-informed care*	
Defining trauma	Defining trauma in line with the DSM-5 definitions, and presenting the differences between single event and complex trauma. Discussing the neuroscience and physiological consequences of trauma. Research around trauma and substance use (e.g., Adverse Childhood Experiences research), and the effects of trauma on health.
Secondary and vicarious trauma	Recognizing vicarious and secondary trauma in AOD staff.
Introduction to trauma-informed care	Covering the five principles of trauma-informed care. Discuss how TIC is beneficial for both staff and clients. Considerations of TIC for different minority and vulnerable groups (e.g., culturally and linguistically diverse peoples), with reference to intergenerational trauma.
*Advanced residential-specific skills*	
Unpacking complex trauma	Discussion of factors than affect recovery such as developmental stage, support, stress, pre-morbid functioning, and type of trauma. The role of disrupted attachment and early interpersonal trauma that will be prevalent in AOD residential clients.
The consequences of complex trauma	Discussing the consequences of trauma on emotions and cognitions, and how this can present following single incident versus complex trauma, including: emotion dysregulation, maladaptive coping styles, negative beliefs about self, disconnection from the body, interpersonal difficulties.
Understanding challenging behaviors	Teaching staff to identify potentially challenging behaviors in clients and understand why they may be present in specific contexts.
Symptoms that can present as a result of trauma	Training to identify potential symptoms of trauma history and facilitate understanding of the function of these for clients, including: suicidality and self-harm (thought/plan/intent to deliberately self-harm or die by suicide), psychosis (hallucinations, delusions, grossly disorganized behavior), dissociation (transient periods of cognitive-perceptual disturbance in response to stress).
Managing and presenting vicarious trauma	Symptoms of vicarious trauma, and how these may arise in certain work contexts or to certain people (i.e., vulnerability factors). Facilitating staff to identify vicarious trauma through five recurring factors. Organizational strategies to prevent or alleviate vicarious trauma. Tips for practice and screening tools for staff.
PTSD screening in clients	Staff understanding of the importance of screening for PTSD in clients, and how to administer and interpret this.
Conversations on trauma	Teaching staff skills on talking about trauma with clients and how to contain disclosures of trauma.
Managing triggers and distress	Teaching staff how triggers for clients can lead to emotion dysregulation and how to deal with this, including: how to respond in the moment, creating a sense of safety for clients, grounding exercises.
Responding to trauma symptoms	Teaching staff on how to respond to: suicidal ideation, triggers, psychotic experiences, dissociation, aggression, challenging behaviors, conflicts between clients.
Importance of formulations	Using formulations for clients to help inform treatment, particularly when challenging behavior arises.
What TIC looks like in practice	Looking at what the organization is currently doing that is trauma-informed, and what changes need to be made to make it more trauma-informed, and discussing potential barriers to this. How AOD services can inadvertently retraumatise clients and how to minimise this.

Clinical supervision in the new TIC model following the workshop will be provided to staff involved in direct client care, to help consolidate their knowledge, skills, and confidence in TIC. The process of supervision will involve support in the new model and critical reflection through the lens of TIC principles. Weekly supervision will involve a 1:2 ratio of supervisor to supervise for 40 min each week. This will be provided by a clinical psychologist and member of the project team during the initial implementation of the TIC model. Once the formal evaluation has finished, period, supervision will be provided by a team leader/ manager within the service who has received training from a clinical psychologist in TIC supervision practices.

#### The service environment

The procedures, practices, and physical setting of the residential facility were reviewed by the research team and staff to target the key TIC principles of safety, trust, choice, collaboration, and empowerment in the service environment. The staff, assisted by the researchers and TIC specialized clinical psychologist developed a poster that highlighted TIC practices at the residential service. Key changes that were made after this review included a staff member allocated to a floor manager position, responsible for monitoring and managing client behavior and the day-to-day activities of the service, including adherence to the program schedule.

Staff will be provided with trauma-informed guidelines to reinforce the training content. The guidelines include examples of how to discuss trauma with clients, preparing for and managing trauma disclosures, strategies for creating a safe environment (e.g., speaking in soft and gentle tone, attending to non-verbal’s, being aware of body and positioning, normalization, and validation practices), and example scripts and strategies for managing and preventing distressing trauma-related responses in clients. Additionally, staff will be provided with information on taking a strength-based approach (rather than a deficit model), supplemented with resources for staff and clients, and posters on site.

#### The group treatment program

The existing 6-week therapeutic group program was extensively revised by the research team with staff to create a more structured, consistent, and trauma-informed approach, described below. Three one-day training workshops on the new program were delivered by the research team to residential staff across 2020 and 2021. Staff were encouraged to provide feedback on the program throughout training to further refine the content. Staff who are in the role of treatment facilitators will deliver the 6-week therapeutic program.

Clients entering the service will take part in a 1 h introductory group, developed to: (i) provide clients with a general overview and feedback on their routine Outcome Measures collected at treatment entry; (ii) enhance clients’ motivation to complete residential treatment using a motivational interviewing framework (e.g., completing decisional balance form on staying versus leaving residential facility); and (iii) understanding the benefits and skills they will practice during the residential program.

Clients will take part in ‘Grit’, an 12-session strength-based, self-regulation and wellbeing program (62); ‘Healthy Recovery’, a 6-session intervention targeting health behaviors for people in treatment for SUDs (63); and ‘ReFrame’, a 12-session Cognitive-Behavior-Therapy (CBT) intervention for substance use. The content of each intervention is summarized in the Supplementary Tables S1–S3. Additional group sessions may also be conducted outside of this program, which clients can attend if the content is relevant to their needs and goals (e.g., parenting group, gambling group, Alcoholics Anonymous).

##### Trauma-informed components

Psychoeducation on the impacts of trauma and links with SUDs was integrated into the three group interventions. Specifically, Grit was adapted to include sessions exploring of the impacts of long-term stress (including trauma) on the body, how early stress and adverse childhood experiences can impact relationships and schemas of the world, and coping skills designed for managing strong emotions arising from stress. The link between trauma and adverse childhood experiences and substance use was also embedded into the program content. The content of the program was also evaluated to ensure its consistency with TIC, through ensuring that: (i) there were no aggressively confrontational practices, exercises or discussions in the manuals that may be triggering for clients, (ii) staff could provide optional adaptations to the delivery of traditionally therapeutic exercises that may be difficult for clients with a trauma history (e.g., mindfulness activities that focused on body awareness), (ii) in-the moment-strategies provided if clients did experience distress in the sessions (e.g., distress tolerance exercises), in addition to preventative strategies to reduce the likelihood of distress arising, and (iii) clear boundaries and expectations stated at the start of each session.

Additionally, all group sessions across the three interventions were evaluated to ensure consistent language is used and attuned to clients’ varying levels of literacy. Consistent and accessible language for clients to understand and consolidate learnings from the groups is important for empowerment, a critical component of TIC. In addition to consistency in language, a strengths-focused approach was implemented across the program, where clients’ strengths are emphasized and strength spotting in oneself and others is woven throughout the therapeutic program and other activities. This included the addition of one formalized strength-spotting group session a week where clients were encouraged to identify and provide strengths feedback to each other.

To ensure TIC practices are consistently applied across the different therapeutic interventions, a section on trauma-informed practices was added to the Grit, Healthy Recovery, and ReFrame manuals. This was designed to reinforce the strategies of creating a safe group environment, providing consistent language for concepts discussed with clients, and reinforcing the skills discussed in staff training and in the revised policies, procedures, and guidelines (outlined in The service environment section).

#### Trauma screening and feedback

Routine screening for trauma was conducted at service entry using the Primary Care PTSD Screen for DSM-5 (PC-PTSD) (64) as part of the routine measures collected by the service. Routine measures are collected using an online survey that is sent to participants once a client is entered into client-relationship management system by staff. The five-item PTSD-PC screens for PTSD symptoms over the last month, among individual who have experienced a traumatic event in their lifetime. Staff will discuss Primary Care PTSD Screen scores with clients in their first one-on-one case management session, typically occurring in the first week at the service, so that clients are provided timely and appropriate feedback on their symptoms. As outlined in Workforce development section, staff will receive training in understanding and delivering feedback on PTSD symptoms and will have ongoing supervision in this area.

#### Trauma-informed therapy

Clients with a positive screen on the PC-PTSD are assessed for symptoms of PTSD on the PCL-5 (65) with those with elevated symptoms (a score of 27+) then considered for eligibility to receive Cognitive Processing Therapy (CPT). Exclusions for the CPT component are: (a) non-fluent in English, (b) acutely suicidal, (c) a current diagnosis of schizophrenia, (d) currently experiencing a manic episode, (e) an intellectual disability that does not allow for the comprehension of the CPT material, and (f) experienced an index early childhood trauma that occurred before the age of three. Eligible clients will be offered 10 × 90 min sessions of CPT (66) delivered in-person or via telehealth/videoconference while they are in residential treatment. Clients who are discharged from the service will be offered CPT via telehealth if they have completed at least 2 sessions. The 10 sessions of CPT will be delivered twice a week over 5 weeks, to fit with the 6-week residential program [Table 2; (44)].

**Table 2:** An overview of the 10-session CPT content.

Week	Session	Content overview
Week 1	N/A	Routine screening and consenting
Week 2	Session 1	Introduction to CPT, psychoeducation, CPT overview
	Session 2	Introducing thoughts, feelings, and identifying stuck points
Week 3	Session 3	Processing events, thoughts, and feelings
	Session 4	Level of responsibility and introducing challenging questions
Week 4	Session 5	Processing challenging questions and introducing patterns of problematic thinking
	Session 6	Processing patterns of problematic thinking and introducing challenging beliefs
Week 5	Session 7	Reviewing challenging beliefs, introducing safety and trust issues
	Session 8	Processing safety and trust issues and introducing power and control issues, and esteem issues
Week 6	Session 9	Processing power and control issues and esteem issues, introducing intimacy issues
	Session 10	Processing intimacy issues, identifying future care pathways, and identifying unprocessed stuck points

Sessions were approximately 90 min long to ensure coverage of content and were scheduled with at least 3 days apart, to allow for practice between sessions. Each session was accompanied with home practice tasks where clients are asked to complete activities that map onto the concepts taught from the corresponding therapy sessions. The PCL-5 was collected at the beginning of every session to monitor symptom changes.

The CPT component of the TIC model will be delivered by three study authors (VM, NK, ZW) with a capacity limit of 8 clients receiving treatment simultaneously. Clients will commence CPT in their second week of treatment (to allow time to settle into the service and ensure clients are no longer experiencing symptoms of withdrawal). Clinicians will be trained in CPT by completing a recognized 13-h online course (Medical University of South Carolina, 2021), followed by a two-day training workshop and supervision on a weekly basis for 26 weeks delivered by a certified CPT training provider.

## Procedure

The TIC model was developed over a 6-month period during 2020, and the initial implementation, including workforce training, occurred post development of the program. All clients will receive the residential TIC model as part of routine care. The evaluation phase of the TIC model will occur over a 12-month period once the initial implementation has been completed. Client outcomes will be assessed at baseline and 1-, 3-, 6- and 12-months follow up (till December 2022). Within 1 week of entry to the service, clients will receive verbal and written information of the trial by a research assistant and informed consent will be obtained. Only the data of clients who consent to participate in the project will be used to evaluate the effectiveness of the new model. All client participants will be followed up for intent-to-treat purposes, regardless of treatment completion. Reminders to complete the survey will be sent to participants via SMS and email. At 1-month and 3-month timepoints, the online survey and reminders will be sent as part of the service’s usual Outcome Measures procedure. At 6- and 12-months, research assistants will send out the surveys (as these time-points are not part of the services usual outcome measure procedures). Participants will be followed-up over the phone by research assistants not involved in treatment delivery or model design, if the survey is not completed within 1 week. Participants will be reimbursed $20 for completing each survey.

Staff outcomes will be collected at the TIC training, and again at 3-, 6-, 12- and 18-months post-training, via online surveys sent by email from research assistants not involved in treatment delivery or TIC model design. Staff will be sent reminders to complete the surveys by these research assistants and general reminders regarding the study by their team leader. Staff who are no longer working at the service will not be followed up.

The sustainability of the TIC model will be assessed for a 12-month period after the evaluation phase is complete, to determine if delivery of the TIC model is sustained once the formal evaluation ends. All clients who enter the service consent to their deidentified data being used for service evaluation purposes. During this time, the researchers will have minimal involvement in the running of the model. Service outcomes will be collected during this period (see below for outcomes).

### Sample size calculation

A power calculation for a single-arm, repeated measure design over four time-points indicate we will require 60 clients for adequate power (0.9) to detect a moderate effect (*f* = 0.25) for primary client outcomes (estimating an error < 0.01 to correct for multiple outcomes). Given our recruitment period of 12-months, we expect an actual sample size of approximately 177 clients, and a retention rate of 81% (*n* = 143) at 1-month, 74% (*n* = 130) at 3-months, 64% (*n* = 113) at 6- and 12-months. This estimate was calculated by the recruitment rate for a similar therapeutic study held at the same facility (62). The staff sample size is determined by the number of staff at the participating facility, approximately 20 people.

### Outcomes

#### Client outcomes

Client outcomes will be collected upon entry to the residential treatment service (baseline), and again at 1-, 3-, 6-, and 12-months later. See Table 3 for the list of measures, including measures that are collected as part of routine care and the additional measures completed for the evaluation. The primary outcome for the evaluation will be substance-related outcomes, assessed via the World Health Organization Alcohol, Smoking and Substance Involvement Screening Test (ASSIST) (67) and the Australian Treatment Outcomes Profile (ATOP) (68). Quality of life is assessed by the relevant items on the ATOP. PTSD upon treatment entry is measured using the Primary Care PTSD Screen for DSM-5 (64), a brief five-item measure of symptoms of PTSD over the last month. Those with a positive screen on the PC-PTSD (scored 3+), will be asked to complete the PTSD Checklist (PCL-5) (69), a 20-item measure of PTSD symptom severity, which will be the primary outcome for the CPT analyses. Secondary outcomes include measures of depression and anxiety using the Patient Health Questionnaire (PHQ-9) (70), (9-items) and the Generalized Anxiety Disorder Scale (GADS-7) (71) (7-items), respectively. Client satisfaction will be assessed via the five-item Patient Experience Questionnaire from the Improving Access to Psychological Therapies program (72). Open ended responses will also be included to collect feedback from the participants about their level of satisfaction with their treatment and to better inform treatment procedures.

**Table 3:** Study timelines and outcomes.

	Enrolment		Treatment		Follow up			
TIMEPOINT	*-t_1_*	*-t_2_*	*t1*	*t2*	6 weeks	3 months	6 months	12 months
*ENROLMENT:*								
*Staff informed consent*	X							
*Client informed consent*		X						
*Client eligibility*		X						
*INTERVENTIONS:*								
*Model development*	X							
*Staff training*		X						
*TIC trial*				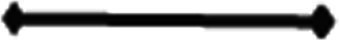				
*Sustainability*					X	X	X	X
*Evaluation:*								
*Client measures*		X			X	X	X	X
Demographics		X						
WHO-assist		X				X	X	X
ATOP		X			X	X	X	X
PHQ-9^a^		X			X	X	X	X
GAD-7		X			X	X	X	X
PTSD screen*		X			X	X	X	X
PCL-5*		X			X	X	X	X
Distress tolerance scale		X			X	X	X	X
Resilience evaluation scale		X			X	X	X	X
PEQ					X	X	X	X
*Staff measures*	X				X	X	X	X

^a^PHQ-9 includes an item on suicide ideation. If suicide ideation is endorsed daily on this item, risk management processes are followed. *Clients complete only if they report experiencing a traumatic event in their lifetime. WHO-ASSIST, World Health Organization Alcohol, Smoking and Substance Involvement Screening Test; ATOP, Australian Treatment Outcome Profile; GAD-7, Generalized Anxiety Disorder; PHQ-9, Patient Health Questionnaire; PTSD, Post Traumatic Stress Disorder Screen, PCL, PTSD Checklist; PEQ, Patient Experiences Questionnaire.

#### Staff outcomes

Staff outcomes will be collected at the TIC training (baseline), and again at 3-, 6-, 12- and 18-months post-training. At each time point, the 59-item Knowledge, Principal Support, Self-efficacy, and Beliefs that Predict Commitment to Trauma-informed Care Survey (73) will be used to measure commitment to TIC (6 items), support for TIC (6 items), self-efficacy to implement TIC (7 items), beliefs about trauma in the workplace (10 items), and foundational knowledge about trauma (30 items). Staff perceptions of the five TIC values within an organization are also measured using the 10-item Trauma-Informed Climate Scale-10 (TICS-10) (74). Workplace satisfaction, burnout, and fatigue are assessed using the 30-item Professional Quality of Life Scale (V5, ProQOL) (75). An Organizational Self-Assessment of TIC (76) will also be measured at each time point to assess the extent to which the organization is implementing and progressing with a TIC model in three domains: organizational readiness for trauma-informed care change (6 items), competent trauma-informed organizational, clinical, and milieu practices (11 items), and consumer and family engagement (8 items). We will also measure staff turnover rates, and supervision attendance.

#### Service-level outcomes

Outcomes at the service-level will be evaluated to determine the feasibility of and adherence to the model throughout the trial (approximately 24 months). Specifically, we will examine number and percentage of clients completing the 6-week cycle, admission and readmission rates, number of discharges and reason for discharge, average length of stay, number of individual sessions received while in treatment, time taken to receive first session, group session attendance rates, and percentage of group sessions run as per program.

#### Fidelity

Fidelity of the model will be assessed via multiple methods. The fidelity of the group program will be assessed via completion of group attendance lists, and by session checklists completed by treatment facilitators for group sessions run during a 6-week cycle of the model. Additionally, during this time, session checklists will be completed by independent observers, and inter-rater reliability checks will be conducted. To assess the fidelity of the individual CPT component of the model, clinicians will audio record sessions. A random sample (20%) of session recordings will be independently rated for treatment fidelity to ensure the core features of the relevant CPT session are delivered.

### Data and analyses

Data integrity will be checked through a variety of methods, including examining valid values, range checks, and missing values analysis. Acceptability and feasibility of the model will be determined by analyzing frequencies of the outcomes outlined above, during both the evaluation and sustainability phases of the trial.

The primary evaluation will examine within subject change of client outcomes, using generalized linear mixed models, including confidence intervals and effect sizes (77). For client outcomes, we will examine change in primary and secondary outcomes comparing baseline to 3-, 6-, and 12-month follow-ups. Analyses will be conducted by authors ZW and MC, using intention-to-treat approach, with all baseline data included for those who were eligible and consented to take part in the trial, as well as subset analyses examining participants who received TIC model only (i.e., did not receive CPT). To assess the outcomes of the CPT component of the trial, we will assess participants who received CPT compared to matched-pair controls of participants completed TIC alone (trauma exposure, age, gender, baseline severity) using mixed effects model repeated measures. The mixed method approach included time (baseline, 3-, 6-, 12-months), group (intervention: TIC+ CPT, control: TIC), and a time x group interaction as fixed effects. We will also perform exploratory analyzes, including (a) using Bayesian methods to assess potential changes, as this form of analysis is less reliant on value of *p* testing, and (b) exploring the interaction between SUD and PTSD symptoms. Change over time in staff and service outcomes will also be examined and within-subject and differences at each time-point change over time will be reported.

## Ethics, adverse events, and risk management processes

The study has received Human research ethics approvals by the University of Queensland (Approval number: 2020000949) and has been approved by the treatment service and the broader service organization. All participants will have substance use problems, with likely SUDs and other mental health comorbidities. Increased vulnerability and decreased social engagement must be considered, however the project has been specifically designed to support complex and typically underserved populations. As the project will be held within a residential setting, clients will have 24-h access to support, and are connected with a community worker upon leaving the program. Any safety or urgent treatment issues will be managed as per usual safety and risk management procedures of the residential treatment facility. These processes include assessing for suicide and self-harm risk on entry, development of risk management/safety plan with caseworker, and ongoing monitoring of risk. If clients do present at imminent risk of harm or other adverse events, caseworkers will work with the clients, utilizing the client’s personalized risk management plans, including hospitalization if required. Clients learn distress tolerance skills for managing difficult emotions as part of this as part of the 6-week TIC program. Further, all therapists will be trained clinicians, experienced in managing risk, distress, and discomfort in clients.

Client’s suicide risk will be assessed at follow-ups via the Outcome Measures. If participants report suicide ideation in the past 2 weeks at 1- or 3-month timepoints, the LLW case-manager for the client will instantly be informed through a client-management system, will conduct a risk assessment, and provide the client with counseling and community support services as required. For clients who are no longer active with Lives Lived Well, the risk assessment and subsequent required supports (such as connecting to community services) will be conducted by a psychologist from the research team.

Staff will be able to report adverse effects and subsequently provided with support via weekly supervision and as needed by their team leader.

The project research team meet regularly with team leaders and area managers of the service involved in the project, as well as meet monthly with organization management. At these meetings, feedback is invited on the conduct of the research and research progress is provided to the organization, as well as soliciting incidents of adverse effects. Additionally, spontaneous participant or clinician reports of adverse effects will be recorded. The model and research will be adapted in response to feedback, if required.

## Dissemination

The findings will be disseminated to treatment services and through conferences and publications in scientific journals. Data will be collected and stored electronically in a secure data file, only accessible by the research team. De-identified research data may be used as comparative data in future projects for secondary analysis with the consent of participants.

## Conclusion, strengths, and limitations

This study details a model of trauma-informed care for residential substance use treatment that was co-developed with key stakeholders. The new model includes workforce development, ongoing supervision for staff, the inclusion of trauma screening and feedback, psychoeducation and coping strategies; integrated trauma-informed care into a 6-week group program, and the provision of specialist trauma-focused therapy. We will examine client, staff, and service outcomes over 12 months post implementation; and the sustainability of the delivery of the model will also be examined. Strengths of the current study include this holistic and codesigned approach, the use of validated measures across individual (micro), staff (meso), and organization (macro) levels, and examining outcomes over a 12-month period. The use of a single-arm uncontrolled design conducted at one site means no conclusions can be made about the comparative benefits of trauma-informed care over usual treatment and the generalizability of the model to other service settings. Further, research team was involved in the development of the model and the delivery of aspects of the model, and also subsequently will be involved in the collection of outcome measures and analysis. This design can affect the objectivity of data collection and analysis. Participants may alter their behavior or responses, consciously or unconsciously, based on their awareness of the researchers’ role and expectations, leading to response bias. Additionally, researchers may unintentionally interpret or analyze data in a manner that aligns with their preconceived notions or desired outcomes, introducing confirmation bias. To mitigate these risks, outcome measure will be collected online where possible and by an independent research assistant via phone, and we will ensure transparent reporting of methods, data handling, and potential conflicts of interest, to enhance the trial’s transparency and reproducibility.

## Author contributions

MC and ZW led the writing for the protocol. NK, VM, RN, VC, and LH provided edits, comments, and oversight. All authors were involved in the development of the protocol, contributed to the article, and approved the submitted version.

## Funding

This implementation trial is funded by a Drug and Alcohol Program grant over 2019–2022 awarded to the Chief Investigator Professor Leanne Hides, National Center for Youth Substance Use Research (NCYSUR), by the Australian Government Department of Health. The project forms part of an approved research program funded to deliver novel, high-quality research on AOD use under the Department of Health AOD Research Grants funding scheme. The AOD Research Grants scheme operates within the Australian Government’s Drug and Alcohol Program, as part of Outcome 2 – Health Access and Support Services, Program 2.4 – Preventive Health and Chronic Disease Support.

## Conflict of interest

The authors declare that the research was conducted in the absence of any commercial or financial relationships that could be construed as a potential conflict of interest.

## Publisher’s note

All claims expressed in this article are solely those of the authors and do not necessarily represent those of their affiliated organizations, or those of the publisher, the editors and the reviewers. Any product that may be evaluated in this article, or claim that may be made by its manufacturer, is not guaranteed or endorsed by the publisher.
