# Supplementary material for: Study protocol: implementing and evaluating a trauma-informed model of care in residential youth treatment for substance use disorders
Source: Front Psychiatry. 2023 Sep 27;14:1169794. doi: 10.3389/fpsyt.2023.1169794 (PMC10572352; doi:10.3389/fpsyt.2023.1169794)
Supplement: Supplementary file 1 [file Data_Sheet_1.PDF]

Table S1: Core components of the Grit intervention

Session Number and Name		Wellbeing Target	Content	Reference
1	Learning to Breath-Ground-Centre	Emotional; Psychological; Physical	Clients learn practical methods to ground themselves and remain in the present moment. The sessions has a particular emphasis on breathing techniques, grounding methods, and ways to identify their centre or core.	Rock and Water program (Raymond, 2005; Ykema, 2002)
2	Focus/Mind Fit	Psychological; Physical	Clients learn the final part of "being present", which is focussing. They also experience up to four practical mindfulness tasks (i.e., a guided meditation, mindful boxing, mindful eating, mindful walking).	Rock and Water program (Raymond, 2005; Ykema, 2002); mindfulness-based relapse prevention programs (Bowen, Chawla, & Marlatt, 2011; Roos et al., 2019).
3	Stressful Life Events	Emotional, Psychological, Physical	Clients learn the impact of stressful life events on their emotions and body. They also learn ways of identifying, and containing emotions that can arise due to stress, and ways to articulate these to others.	Siegel, D. J., & Solomon, M. F. (Eds.). (2003). *Healing trauma: Attachment, mind, body and brain (Norton Series on Interpersonal Neurobiology)*. WW Norton & Company.
4	Social Supports	Social	Clients learn the importance of social support, how our early life experience can impact our relationships and the way we connect with others, and the different types of connections we can have.	Groups for Health program (Haslam et al., 2019; Haslam, Cruwys, Haslam, Dingle, & Chang, 2016), and used in other residential AOD treatment settings (Best et al., 2016; Dingle et al., 2020; Mawson et al., 2015).
5	Mapping your Social World	Social	Clients complete an exercise to map out their social world (social identity mapping task), including identifying importance, positivity, level of support, representativeness, and compatibility of their social connections.	Groups for Health program (Haslam et al., 2019; Haslam, Cruwys, Haslam, Dingle, & Chang, 2016), and used in other residential AOD treatment settings (Best et al., 2016; Dingle et al., 2020; Mawson et al., 2015).
6	Character Strengths	Social; Psychological	This session begins with group exercises to draw out character strengths. Clients then learn how to identify their own strengths and the strengths of others, and identify what strengths they would like to build and develop on their recovery journey.	Strengths-based mindfulness approach (Niemiec, 2012)
7	Social Wellbeing	Social	Clients identify barriers to group belonging and connecting with others, and areas of compatibility/incompatibility and loss in their social networks. Clients also learn the importance of giving and receiving support in relationships.	Groups for Health program (Haslam et al., 2019; Haslam, et al., 2016), Social Identity Mapping (Best et al., 2016; Dingle et al., 2020; Mawson et al., 2015).

8	Identity and Values	Psychological	Clients identify important parts of their self-concept and examine how their social connections and strengths represent those aspects of themselves. Clients also engage in an exercise identifying their values and identify how much they are living in alignment with their values.	ACT Approach - (Hayes, Strosahl, & Wilson, 1999; Harris, 2013)
9	Communication	Social; Psychological	Clients learn about the different communication styles (passive, aggressive, and assertive); strategies for effective communication; how to be assertive; and, tips on having difficult conversations. Clients also examine their own and others' boundaries, and ways to establish boundaries.	Developed for Grit
10	Emotions and Music	Emotional	Clients learn the connection between music and emotion, using music to identify a range of emotions. Clients also learn how to mindfully experience emotions and strategies to enhance joy and to savour positive experiences.	Tuned In music emotion regulation program (Dingle & Carter, 2017; Dingle & Fay, 2017; Dingle, Hodges, & Kunde, 2016); Music app (Music eScape; Hides et al., 2019).
11	Emotional Wellbeing	Emotional	Clients learn factors that underlie and impact their experience of emotion, understand the impact of substance use on emotions, and learn how to improve and manage unpleasant emotions by mapping mood and exercise journeys.	Tuned In music emotion regulation program (Dingle & Carter, 2017; Dingle & Fay, 2017; Dingle, Hodges, & Kunde, 2016); Music app (Music eScape; Hides et al., 2019).
12	Healthy Me	Emotional; Psychological; Physical	This session revises the breathe-ground-centre-focus techniques, before focusing on trivia about physical activity, sleep and diet; and in particular, how these are impacted by substance use and how they impact on their wellbeing.	Developed for Grit
13	Managing Thoughts and Cravings	Psychological; Physical	The session discusses the connection between thoughts, emotions, physical sensations, and behaviours. Clients learn strategies to manage unhelpful thoughts and cravings using mindfulness and breathe-ground-centre-focus exercises.	Mindfulness-based relapse prevention programs (Bowen, et al., 2011; Roos et al., 2019);
14	Difficult moments and developing Goals	Emotional; Social; Psychological; Physical	Clients learn practical strategies to aid them in managing conflict and dealing with difficult situations. They will also draw upon key components learnt throughout the program to develop goals through their recovery journey. Facilitators acknowledge individual client strengths and graduating clients.	Mindfulness-based relapse prevention programs (Bowen, et al., 2011; Roos et al., 2019); Straight ahead: Transition skills for recovery. A Training Manual from the TCU/DATAR Project (Bartholomew, Simpson, & Chatham, 1993)

Table S2: Core components of the adapted Healthy Recovery program

Session Number	Health Target	Content
1	Overview	Introduction to the 4 key principles of health (healthy eating, non-smoking, exercise and physical activity, and sleep). Clients also receive psychoeducation on the health advice and discuss why health is important and the challenges and benefits of becoming healthier
2	Smoking	Clients discuss the non-smoking policy in the residential facility and receive psychoeducation on the impact of nicotine on the brain and mental health. The clients complete an exercise on the costs of smoking, and the motivations and benefits to change, and develop a change plan for smoking.
3	Healthy Eating	Clients discuss why healthy eating is important and factors that influence food intake. This is followed by a discussion on the positive and negative impacts of healthy and unhealthy eating and water and health. Following this, complete a trivia exercise on water intake, and develop a change plan for healthy eating.
4	Physical Activity	Clients discuss why physical activity is important in recovery and take part in an exercise on what is moderate and vigorous activity. The clients then take part in an exercise of thinking back to a time they enjoyed exercise, and a discussion on challenges to maintaining physical activity. The clients then develop a goal and change plan.
5	Sleep	Clients identify why sleep is important in general and in recovery, followed by psychoeducation on stages of sleep and circadian rhythm. The clients than engage in a group activity on Myths about sleep, followed by a discussion on ways to improve sleep. The session concludes with clients develop a change plan.

Table S3: Core components of the ReFrame intervention

Session Number and Name		Wellbeing Target	Content	Reference
1	Motivating Change	Motivational enhancement	Clients learn about motivation, expanding awareness of various forms of motivation and how these influence what we think, feel, and do. Motivations for making or not making a change with substance use are explored, culminating in clients commencing a plan for change.	Motivational Interviewing (Miller & Rollnick, 2013; Naar & Safren, 2017; Wagner & Ingersoll, 2012)
2	Connecting Thoughts, Emotions, Behaviours and Body	Awareness, distress tolerance	Clients learn about the core components targeted in cognitive behavioural therapy; thoughts, emotions, behaviours and body sensations. The links between these components and substance use are explored, with a focus on cravings. The session concludes with clients learning a skill to help manage cravings and other difficult moments.	Cognitive Behaviour Therapy (Hayes & Hofmann, 2017; Hayes & Hofmann, 2018; Hofmann, 2011)
3	Experiencing Emotions	Emotion Regulation and Distress Tolerance	Clients learn about emotions, with a focus on why we have emotions and how we experience emotions. The impact of substances on emotions is discussed, in addition to learning skills to help emotional regulation.	Cognitive Behaviour Therapy, Dialectical Behaviour Therapy, Emotions research (Barrett, Lewis, & Haviland-Jones, 2016; Hofmann, 2011; Pederson, 2015)
4	Stress and Relaxation	Stress, Distress Tolerance	Clients learn how stressful life events impact on the body, and discusses the ‘green’, ‘red’ and ‘blue’ zones of arousal. The role of substances as an attempt to regulate states is discussed. Three core strategies are learnt and practiced to assist with relaxation and arousal reduction.	Cognitive Behaviour Therapy (Hayes & Hofmann, 2018; Hofmann, 2011)
5	Awareness and Acceptance	Core Mindfulness, Present Moment skills	Clients explore ways to get ‘with it’ and connect with the present moment, and also skills to reduce avoidable suffering through acceptance.	Mindfulness (Linehan, 2014; Pederson, 2013; Bowen, Chawla, & Marlatt, 2010)
6	Helpful and Unhelpful Thoughts	Thought monitoring, cognitive distortions	Clients learn about thoughts and styles of thinking, including how substances influence thoughts. Helpful and unhelpful styles of thinking are discussed, including exercises to identify common thinking traps.	Cognitive Behaviour Therapy (Hayes & Hofmann, 2017; Hayes & Hofmann, 2018; Hofmann, 2011)
7	Reframing	Managing unhelpful thoughts	This session covers the core CBT skill of cognitive restructuring (Reframing) in detail, including strategies for challenging unhelpful thoughts and creating more helpful thoughts to replace them.	Cognitive Behaviour Therapy (Hayes & Hofmann, 2017; Hayes & Hofmann, 2018; Hofmann, 2011)

8	Values into Action	Identity, goal setting	Clients learn about values and goals, and are engaged in an exercise to identify and clarify some of their core values. This is complemented by learning about how to set effective, values-informed goals.	Acceptance and Commitment Therapy, Cognitive Behaviour Therapy (Hayes & Hofmann, 2018)
9	Planning for Action	Routine and Structure	Clients learn about the importance of planning and routine, and balancing needs and wants, structure and flexibility, and predictability and novelty.	Dialectical Behaviour Therapy (Linehan, 2014; Pederson, 2013; Pederson 2015)
10	Problem Solving	Problem Solving and Interpersonal Effectiveness	Clients engage in experiential exercises of problem solving and learn a structured approach for tackling tricky situations relating to AOD use.	Dialectical Behaviour Therapy (Linehan, 2014; Pederson, 2013; Pederson 2015)
11	Relapse Prevention	Relapse Prevention	Clients learn about the difference between a lapse and relapse, and how to identify if they might be at risk of returning to substance use, and managing high risk situations. Additionally, clients learn skills for getting back on track after a lapse, to prevent a lapse turning into a relapse.	Cognitive Behaviour Therapy and Dialectical Behaviour Therapy (Hayes & Hofmann, 2018; Hofmann, 2011; Linehan, 2014; Pederson, 2013; Pederson 2015)
12	Burning and Building Bridges	Relapse Prevention	Using the metaphor of 'bridges', clients learn about connections in their lives that support or hinder recovery, focusing on people, places, things, beliefs and habits. Clients are encouraged to identify bridges they need to 'burn', and bridges they want to 'build' to support their recovery.	Dialectical Behaviour Therapy (Linehan, 2014; Pederson, 2013; Pederson 2015)
